# The AMPK-like protein kinases Sik2 and Sik3 interact with Hipk and induce synergistic tumorigenesis in a *Drosophila* cancer model

**DOI:** 10.3389/fcell.2023.1214539

**Published:** 2023-10-03

**Authors:** Kewei Yu, Niveditha Ramkumar, Kenneth Kin Lam Wong, Gritta Tettweiler, Esther M. Verheyen

**Affiliations:** ^1^ Department of Molecular Biology and Biochemistry, Simon Fraser University, Burnaby, BC, Canada; ^2^ Centre for Cell Biology, Development and Disease, Simon Fraser University, Burnaby, BC, Canada

**Keywords:** *Drosophila* models of cancer, HIPK, Sik, cancer synergy, imaginal discs, signal transduction

## Abstract

Homeodomain-interacting protein kinases (Hipks) regulate cell proliferation, apoptosis, and tissue development. Overexpression of Hipk in *Drosophila* causes tumorigenic phenotypes in larval imaginal discs. We find that depletion of Salt-inducible kinases Sik2 or Sik3 can suppress Hipk-induced overgrowth. Furthermore, co-expression of constitutively active forms of Sik2 or Sik3 with Hipk caused significant tissue hyperplasia and tissue distortion, indicating that both Sik2 and Sik3 can synergize with Hipk to promote tumorous phenotypes, accompanied by elevated dMyc, Armadillo/β-catenin, and the Yorkie target gene *expanded*. Larvae expressing these hyperplastic growths also display an extended larval phase, characteristic of other *Drosophila* tumour models. Examination of total protein levels from fly tissues showed that Hipk proteins were reduced when Siks were depleted through RNAi, suggesting that Siks may regulate Hipk protein stability and/or activity. Conversely, expression of constitutively active Siks with Hipk leads to increased Hipk protein levels. Furthermore, Hipk can interact with Sik2 and Sik3 by co-immunoprecipitation. Co-expression of both proteins leads to a mobility shift of Hipk protein, suggesting it is post-translationally modified. In summary, our research demonstrates a novel function of Siks in synergizing with Hipk to promote tumour growth.

## Introduction

Homeodomain-interacting protein kinases (Hipks) are conserved kinases that regulate cell proliferation, apoptosis and tissue development through multiple important signaling pathways in development such as Wnt, Hippo, JAK/STAT, Notch, TGFβ, Jnk and Hedgehog (Blaquiere and Verheyen, 2017). Mammals have HIPK1-4, with HIPK2 being the most studied member of this family, while *Drosophila* has a single Hipk ortholog. Aside from important roles in development, dysregulation of HIPK2 is associated with tumour suppression or tumourigenesis depending on the cellular context, as HIPK2 has been shown to bind to a wide variety of transcription factors and cofactors and therefore can modulate their effects through various signalling pathways (Puca et al., 2010; Conte et al., 2023). For example, initial research has been greatly focused on HIPK2 activating p53 through phosphorylation of Ser46, and subsequent promotion of pro-apoptotic activity, therefore HIPK2’s role as a tumour suppressor has been very well-characterized (D’Orazi et al., 2002; Hofmann et al., 2002). However, recent literature has shown that HIPK2 can also act as an oncogene in certain cancers. For instance, HIPK2 copy number gain was found to be associated with non-small cell lung cancer (NSCLC) cell lines through activation of the Yes-associated protein (YAP) pathway and subsequently increased tumour proliferation (Dai et al., 2021). In addition, NSCLC patients with high HIPK2 mRNA expression have a significantly lower five-year survival rate compared with patients with low expression (Dai et al., 2021). This is consistent with previous research in *Drosophila* in which Hipk regulates Yorkie (Yki), an ortholog of YAP, to promote tissue growth (Chen and Verheyen, 2012; Poon et al., 2012). This finding that is upheld across cancer patient data and *Drosophila* research demonstrates that *Drosophila* is a powerful model in which to dissect signaling pathways and gene interactions and that disruptions in normal development can provide powerful insights into disease mechanisms (Verheyen, 2022).

Previously, our lab has characterized a tumorigenesis model based on elevated expression of Hipk in *Drosophila* larval imaginal discs. Imaginal discs are composed of columnar epithelial cells with established apical-basal polarity and thus serve as excellent models for human epithelial cancers. Research from our lab has found that elevated levels of Hipk induce numerous overproliferation and tissue overgrowth phenotypes that are reminiscent of tumorigenesis phenotypes such as hyperplasia and neoplasia (Blaquiere and Verheyen, 2017; Blaquiere et al., 2018; Wong et al., 2019). Hipk-overexpression has also been shown to cause phenotypes indicative of Epithelial to Mesenchymal transition (EMT) such as loss of E-Cadherin, elevation in matrix metalloproteinase-1 (MMP-1) levels, cell migration and metastasis to secondary sites (Blaquiere et al., 2018). In addition, another hallmark of robust Hipk expression is induction of elevated *dMyc* expression, which is a potent oncogene activated downstream of several signaling pathways (Wong et al., 2019). A positive feedback loop between dMyc and aerobic glycolysis was found to support tumor growth in Hipk overexpressing cells (Wong et al., 2019).

While there is a wealth of research focusing on downstream targets of HIPK, there are fewer studies investigating the regulation of HIPK. Vertebrate HIPK2 is tightly regulated by post-translational modifications such as phosphorylation, acetylation and ubiquitination (Saul and Schmitz, 2013). In particular, HIPK2 has been found to be an unstable protein and detectable at very low levels in the cell due to constant degradation by the ubiquitin proteasome system (UPS) (Winter et al., 2008). However, under certain external environmental conditions such as DNA-damage, an upstream regulator of HIPK2, E3 ubiquitin ligase Seven in absentia homolog-1 (Siah-1) is phosphorylated and causes the eventual stabilisation of HIPK2 (Winter et al., 2008). In addition, O-GlcNAc transferase (OGT), which transfers the sugar O-GlcNAc to serine and threonine residues of target proteins, promotes HIPK2 and Hipk stability and induces tumorigenesis in *Drosophila* (Wong et al., 2020). Given the importance of Hipk in development and cancer through numerous signaling pathways, we are interested in how Hipk proteins are regulated and the mechanism of action that results in tumorigenesis. Previously, our lab conducted *in vivo* RNAi screens in *Drosophila* and identified Salt-inducible kinases (Siks) as potential Hipk regulators and in this study, we describe these interactions.

Siks are serine/threonine protein kinases belonging to the adenosine monophosphate (AMP)-activated protein kinase (AMPK) family. While mammals have SIK1-3, *Drosophila* only has Sik2 (mammalian ortholog of SIK1 and SIK2) and Sik3. Most studies of Siks have shown similar cellular functions, so we collectively refer to them as SIKs. SIKs are constitutively auto-phosphorylated and regulate adrenocortical function under physiological changes such as high salt or adrenocorticotropic hormone (ACTH) (Wang et al., 1999). SIKs can be negatively regulated by phosphorylation on conserved residues by PKA (Sonntag et al., 2018). Dysregulation of SIK family members is found in ovarian, breast and prostate cancers (Sun et al., 2020). SIK2 amplification is found in approximately 5% of patients with metastatic breast cancer (Rong et al., 2022). In addition, in the ovarian cancer cell line SKOv3, overexpression of SIK2 was found to induce centrosome splitting by phosphorylating the centrosome linker protein C-Nap1 (Ahmed et al., 2010). Consistently, depletion of SIK2 in mouse xenografts sensitized ovarian cancers to tubulin-targeting drug paclitaxel, identifying SIK2 as a potential target for therapy (Ahmed et al., 2010). In mouse adrenocortical tumour cells, SIK1 is present in both the nucleus and cytoplasm, however when stimulated with ACTH, nuclear SIK is phosphorylated and rapidly translocated from the nucleus to the cytoplasm (Takemori et al., 2002). In addition, research in *Drosophila* has found that Sik2 and Sik3 are upstream regulators of the Hippo signalling pathway, a conserved pathway that is vital for controlling organ size, and Sik2 can increase Yki target gene expression and promote tumorigenesis (Wehr et al., 2013; Hirabayashi and Cagan, 2015). Siks were also found to mediate tumorigenesis via Notch signaling in a combinatorial cancer model involving overexpression of the Notch ligand Delta and loss of chromatin modifiers (Şahin et al., 2020).

Here we use *Drosophila* to investigate novel findings linking Siks and Hipk function in tumorigenesis. Our results indicate that the interaction between both proteins induces significant synergistic tumorigenesis in the wing imaginal disc of *Drosophila* larvae, and regulation of downstream target gene expression involved in growth regulation such as dMyc, Armadillo/β-catenin and the Yorkie reporter *expanded-lacZ.* In addition, Siks also positively regulate the level of Hipk protein in wing imaginal discs. Given the striking synergy in our tumour model, our work may identify vulnerabilities in neoplasias associated with elevated HIPK, SIKs or both.

## Material and methods

### *Drosophila* strains and genetics

Flies were raised on standard cornmeal-molasses food. A *dpp-Gal4/TM6B, Tb Hu* fly line was used to induce transgene expression in larval imaginal discs. The *UAS-Hipk* ^*3M*^ strain was used to overexpress Hipk, since this was used in previous tumorigenic assays and interaction studies (Blaquiere et al., 2018; Wong et al., 2020). Two Gal4 fly lines were used to induce transgene expression: *dpp-Gal4/TM6B* and *expanded-LacZ;Hh-Gal4/SM6A∼TM6B*. Crosses were carried out at room temperature, 25°C and 29°C (indicated in experiment descriptions). Sik2 and Sik3 transgenic strains were obtained from various sources. The following lines were used in this study:

*UAS-Sik2-WT* (untagged wild-type; Wehr et al., 2013)

*UAS-Sik2-CA* (untagged constitutively active Sik2 S1032A; Wehr et al., 2013)

*UAS-myc-Sik2-WT* (tagged wild-type; Choi et al. JBC, 2011)

*UAS-Sik3-WT* (untagged wild-type Sik3-PA; Wehr et al., 2013)

*UAS-Sik3-CA* (untagged constitutively active Sik3-PA S563A; Wehr et al., 2013)

*UAS-myc-Sik3-WT* (tagged wild-type Sik3-PA; Choi et al., PLoS Genetic 2015)

*UAS-Sik2-RNAi* (BDSC #55880)

*UAS-Sik3-RNAi* (VDRC #107458, BDSC #28366, BDSC #39005)

*Sik3-RNAi; Sik2-RNAi* (Note: These RNAi lines were combined in a stock to knock out both Sik genes simultaneously, and is distinct from the previously published *Sik2/3-RNAi* line which targets *Sik2* but affects *Sik3* as an off target (VDRC #103739; Wehr et al., 2013)

In all crosses, Gal4 titration controls were included to ensure that the same number of UAS-driven constructs were expressed. For studies comparing control and experimental larvae, we matched animals based on their developmental stage, and not chronological age (days AEL).

### Imaginal disc dissection, antibody staining and quantification

Imaginal discs from late L3 larvae directly before pupariation were dissected in phosphate-buffered saline (PBS) and fixed in 4% paraformaldehyde (PFA) for 15 min at room temperature. After fixation, samples were washed with PBS with 0.1% Triton X-100 (PBST). After blocking with 5% BSA (Bovine serum albumin) in PBST for 1 h at room temperature, samples were incubated with primary antibodies overnight at 4°C. The primary antibodies used include rabbit anti-Hipk (1:200) (Blaquiere et al., 2018), rabbit anti-dMyc (G077) (1:500, abm), mouse anti-Arm (1:200, DSHB N2 7A1), mouse anti-myc-tag (1:200, Millipore 4A6 05-724) and mouse anti-beta-galactosidase (1:50, DSHB 40-1a). After washing with PBST, samples were incubated with Cy3-and/or Alexa Fluor 647-conjugated secondary antibodies (1:500, Jackson ImmunoResearch Laboratories, Inc.), DAPI (4, 6-Diamidino-2-Phenylindole, Dihydrochloride) (final concentration: 0.2 μg per mL, Invitrogen D1306) for 1 h at room temperature. Samples were mounted in 70% Glycerol/PBS after wash. Images were taken on a Zeiss LSM880 with Airyscan confocal microscope and processed using Zen Blue software or ImageJ.

### Pupal dissection, pharate leg mounting and quantification

To examine developmental delay, flies were allowed to lay eggs for 24 h and the timing of emergence of L3 and pupae were noted. Crosses were kept at 29°C. Pharate pupae were dissected in ethanol and mounted in Aquatex (EM Science). Pharates were imaged in ethanol using the Leica MZ6 modular stereomicroscope and images were processed using ImageJ. Legs were scored for malformation as follows: “severely malformed” legs were those that had lost all tissue architecture in the tarsal segments while ‘suppressed malformation’ legs showed distinct tarsal segments.

### Protein lysate preparation and Western blotting

10 late L3 developmentally stage-matched larval heads (with attached imaginal discs) were dissected and lysed with 1× Cell Lysis Buffer (Cell Signaling Technology), supplemented with 1× Protease Inhibitors (Roche), 1 mM phenylmethylsulfonyl fluoride (PMSF) and 1 mM sodium fluoride (NaF). The tissues were mechanically homogenized and sonicated for 3 × 5 . Lysates obtained after centrifugation for 10 min were stored in a 1× Laemmli buffer at −20°C. Protein lysates were resolved by 8% or 4%-15% SDS/PAGE (at 90 V for 120 min) and then transferred to nitrocellulose membranes (at 20 V for 75 min). Membranes were blocked with 5% BSA in TBST before primary and secondary antibody incubation. Images were acquired by a FujiFilm LAS-4000 Chemiluminescent Scanner. The primary antibodies used: mouse anti-β-Tubulin (1:1,000, abm G098), mouse anti-myc-tag (1:200 Millipore 4A6 05-724). HA-tagged Hipk was detected using HRP-conjugated anti-HA-tag (3F10) antibodies (1:2,500, Millipore Sigma).

### Adult wing mounting and imaging

Adult female fly wings were dissected in 75% ethanol and mounted in Aquatex (EMD Chemicals). Slides were baked overnight at 65°C. The wings were imaged with a Zeiss Axioplan-2 microscope and processed with ImageJ software.

### Co-immunoprecipitation

Protein extracts were incubated with indicated antibodies at 4°C overnight, followed by incubation with a mixture of protein A (Invitrogen 10002D) and protein G (NEB S1430S) magnetic beads for 3 h at 4°C. The beads were washed with lysis buffer and boiled in 4× SDS sample buffer. The supernatants were then analyzed by Western blotting.

## Results

### Depletion of endogenous Siks suppresses Hipk-induced leg malformations

Our lab has extensively studied the effects of Hipk during development and in a tumor model (Blaquiere and Verheyen, 2017; Blaquiere et al., 2018; Tettweiler et al., 2019; Wong et al., 2020; Wong et al., 2020). Expression of Hipk in imaginal discs causes tumorigenic phenotypes including hypertrophy, epithelial to mesenchymal transition and metabolic reprogramming (Chen and Verheyen, 2012; Blaquiere et al., 2018; Tettweiler et al., 2019; Wong et al., 2020). Given these numerous functions, we were interested in understanding factors that can either positively or negatively regulate the activity, stability, or localization of Hipk proteins. Hipks across species are regulated by extensive post-translational modifications, including phosphorylation (Saul and Schmitz, 2013). To characterise potential regulators, we used Hipk overexpression phenotypes and screened through a collection of RNAi lines targeting *Drosophila* kinases, which we had successfully used previously to identify Wnt pathway regulators (Swarup et al., 2015). We hypothesized that if altering the levels of a particular kinase could modify the phenotype caused by Hipk overexpression, then that kinase could be a putative interactor or regulator of Hipk.

**FIGURE 1:**
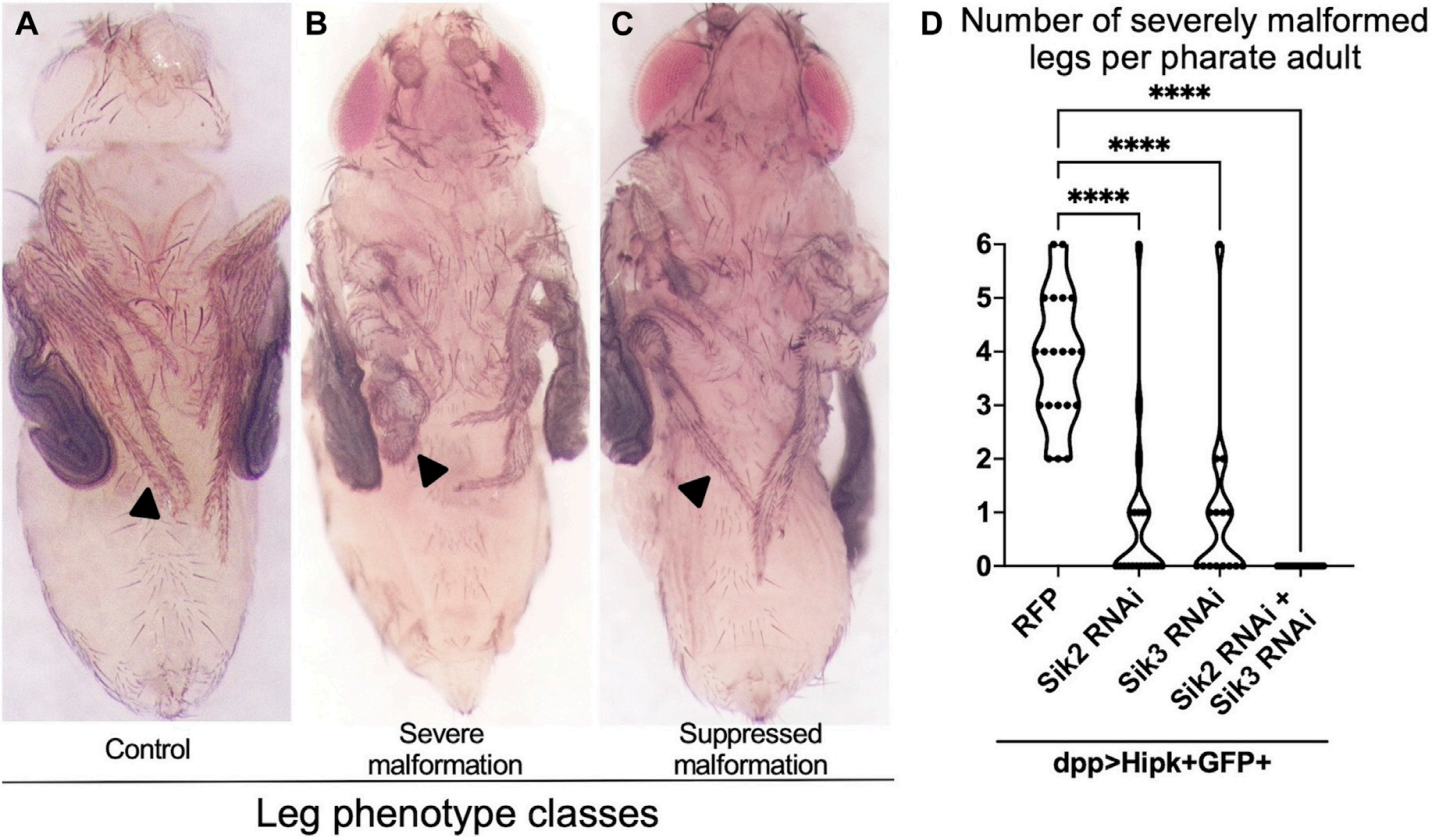
Depletion of Endogenous Siks suppresses Hipk-induced leg malformations **(A–C)** Female adult legs of dpp > GFP **(A)** as control, *dpp > hipk + GFP + RFP* **(B)**, *and dpp > hipk + GFP + sik3-RNAi BDSC #39005* **(C)** *. Arrowh*eads in **(A–C)** mark the tarsal segment of the adult leg, with **(A)** a representative image of a control tarsal segment, **(B)** a representative image of severe tarsal leg malformation and **(C)** a suppressed malformation. **(D)** A violin plot showing the severely malformed leg phenotype per adult of the genotypes *dpp > hipk + GFP + RFP, dpp > hipk + GFP + sik2-RNAi BDSC #55880, dpp > hipk + GFP + sik3-RNAi BDSC #39005 and dpp > hipk + GFP + sik2-RNAi BDSC #55880 + sik3-RNAi VDRC #107458*. Statistical analysis included a one-way ANOVA followed by Dunnett’s test to correct for multiple comparisons to **(B)** *dpp > hipk + GFP + RFP*. *p*-values for the statistical analyses performed correspond to the following symbol: <0.0001(****). *n* = 15–22; 3 biological replicates.

Overexpression of Hipk at 29°C using the *dpp-Gal4* driver causes lethality of the fully formed adult inside the pupal case (known as the pharate stage). Since the wings are extensively folded at this stage, precluding their analysis, we dissected pupae out of their cases to examine the extent of leg malformation, and used this phenotypic readout to assess genetic interactions (Figure 1) (Wong et al., 2020). All pharates examined and quantified were female to eliminate any potential confounding factors arising from phenotypic sex differences. In control pharate adults, all six legs (including the tarsal segments indicated by the black arrowheads) are thin, segmented and tapered (Figure 1A). However, legs in pharates overexpressing Hipk show severe malformations (Figure 1B) that affect between 2 and 6 tarsal segments (indicated by black arrows) of the legs (quantified in Figure 1D). RNAi lines targeting *Sik2* or *Sik3* individually showed a significant suppression of the penetrance and severity of the Hipk-induced malformed leg phenotype (Figures 1C, D), indicating a genetic interaction between Hipk and Siks. When both *Siks* were knocked down simultaneously (Figure 1D), the suppression of overgrowth was stronger than the individual knockdowns. This observation supports our hypothesis that Siks may have redundant, as well as additive effects in Hipk regulation. In subsequent aspects of this study, we focused on Sik2, and data for Sik3 are primarily presented in the supplement since the results were very similar to those found with Sik2, except where noted. These results collectively show that the function of endogenous Siks is required for Hipk to exert its tumorigenic phenotypes in discs, possibly as an upstream regulator needed for activity of exogenous Hipk.

### Elevated expression of Siks and Hipk induce a prolonged third instar larval phase and delayed pupariation

Since we showed that Siks are required for Hipk-induced tumorigenesis, we next sought to investigate phenotypes of flies expressing active Siks together with Hipk. If Siks are needed for Hipk function, we hypothesized that expression of Siks could enhance Hipk activity in a manner like what we have previously shown for the Hipk regulator OGT (Wong et al., 2020). Numerous *Drosophila* tumor models show a paraneoplastic phenotype of an extended larval phase and developmental delay in the tumour-bearing larvae (Bilder, 2004; Menut et al., 2007; Turkel et al., 2013; Doggett et al., 2015; Wong and Verheyen, 2021). Larval tumors continue to grow during this extended phase, resulting in highly neoplastic structures. In some cases, this leads to a delay or failure to pupariate and larval or early pupal lethality. We have previously shown that larvae expressing two copies of a Hipk transgene at 25°C show a delay in pupariation (Wong et al., 2020), though this delay is not observed when using only one copy of Hipk at 25°C. This dose sensitive effect can be mimicked by expressing one Hipk transgene at 29°C, since the activity of Gal4 is greater at higher temperatures (Duffy, 2002). We thus first sought to assess if Hipk and Sik showed effects on larval development by assessing the timing of the larval to pupal transition.

**FIGURE 2:**
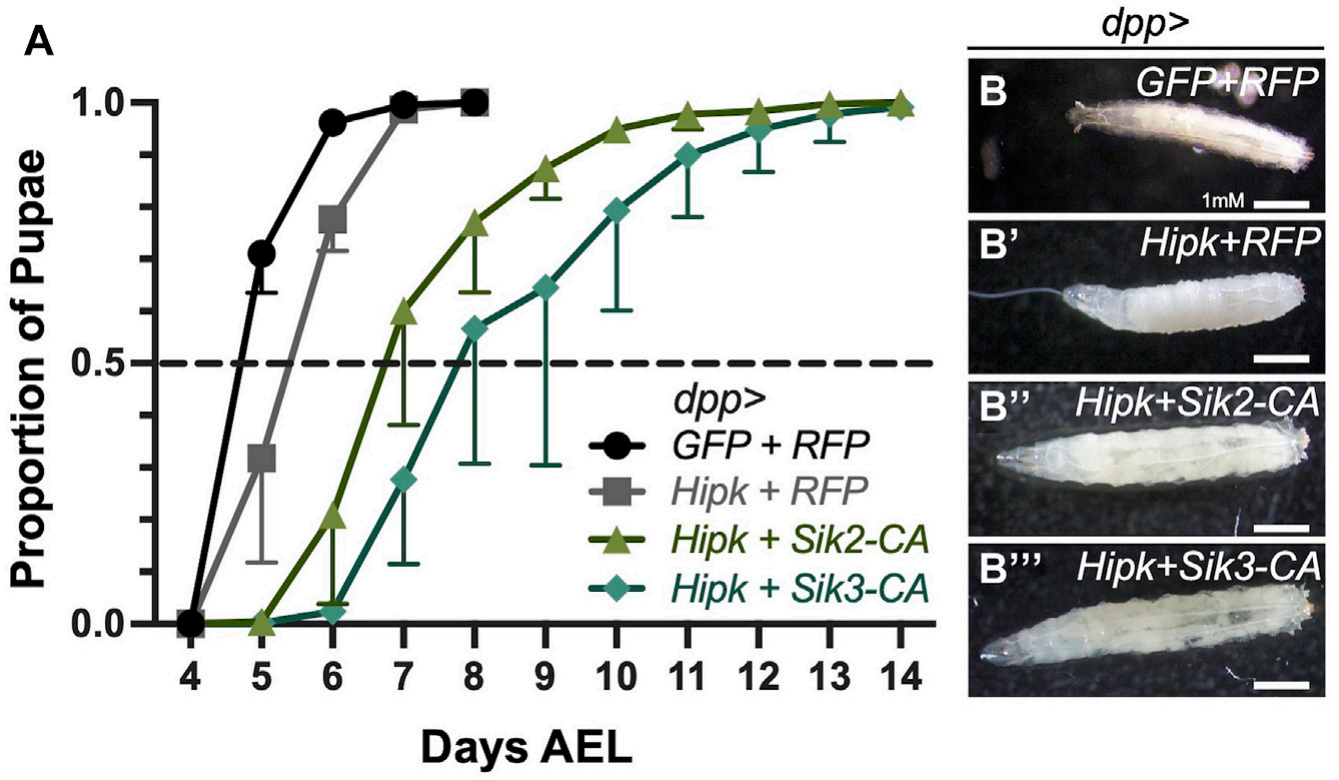
Ectopic expression of Siks and Hipk induce a prolonged third instar larval phase and delayed pupariation **(A)** Developmental timing of larvae expressing the indicated transgenes using the *dpp-gal4* driver. AEL, after egg laying. Values shown are means ± SEM; *n* = 36–121 (pupariation assay), *n* = 4–6 (biological replicate/data point). **(B–B’’’)** Bright field representative images of dorsal views of control (*dpp > GFP + RFP*) and experimental developmentally stage-matched female late third instar larvae for the indicated genotypes (*n* = 7, 10, 10 and 11 respectively). Flies were raised at 29°C.

To monitor developmental timing, we examined progeny from a one-day egg-laying period to ensure synchrony of the offspring. As a control, *dpp-Gal4>GFP/TM6B* was crossed to *RFP* and progeny were incubated at 29°C. Control larvae initiated pupariation at Day 4 after egg laying (AEL), and all larvae have pupariated by Day 8 (Figure 2A). In contrast, *dpp > Hipk + RFP* larvae showed a 1-day delay in rate of pupariation (Figure 2A). This indicates that expression of Hipk induces a developmental delay compared to the control under identical conditions (Wong et al., 2020). To assess the effects of Siks, we expressed constitutively active Sik2 and Sik3 in the *dpp > Hipk* background. Siks are negatively regulated by Protein Kinase A on conserved residues (Sonntag et al., 2018). Mutation of these sites to alanine residues renders the proteins hyperactive and independent of upstream regulation by PKA (Sonntag et al., 2018). We found that progeny expressing *dpp > Hipk + Sik2-CA and dpp > Hipk + Sik3-CA* show a more severe developmental delay where by Day 5 AEL, >50% of control third-instar larvae have pupariated while none expressing *dpp > Hipk + Sik2-CA and dpp > Hipk + Sik3-CA* at the same chronological age had made this transition (Figure 2A). In addition, by Day 8 AEL, 100% of control and *dpp > Hipk + RFP* larvae have pupariated while approximately 20%-30% of *dpp > Hipk + Sik2-CA* and 40%-50% of *dpp > Hipk + Sik3-CA* are still in their larval stage (Figure 2A). Furthermore, a large portion of *dpp > Hipk + Sik3-CA* larvae are unable to pupariate and undergo metamorphosis to become adults, and die as late L3 larvae, while for *dpp > hipk + Sik3-WT* most delayed larvae pupariate eventually (data not shown). Moreover, progeny expressing *dpp > Hipk + Sik2-CA and dpp > Hipk + Sik3-CA* undergo obvious progressive size growth (Figures 2B–B’’’). These phenotypes were not observed when we expressed either Sik2-CA or Sik3-CA transgenes alone under these experimental conditions (data not shown).

### Expression of Siks and Hipk induces significant synergistic overgrowth in third-instar larval wing imaginal discs

Since depletion of Siks could suppress the Hipk-induced leg malformations and co-expression of Siks with Hipk induce a prolonged larval phase that is strikingly like other *Drosophila* tumour models, we investigated the larval disc morphology for evidence of tumorigenic phenotypes. We assessed the proliferation effect of Hipk by quantifying the total size of wing discs as well as the area of GFP-expressing cells, as we have done previously (Chen and Verheyen, 2012; Wehr et al., 2013).

**FIGURE 3:**
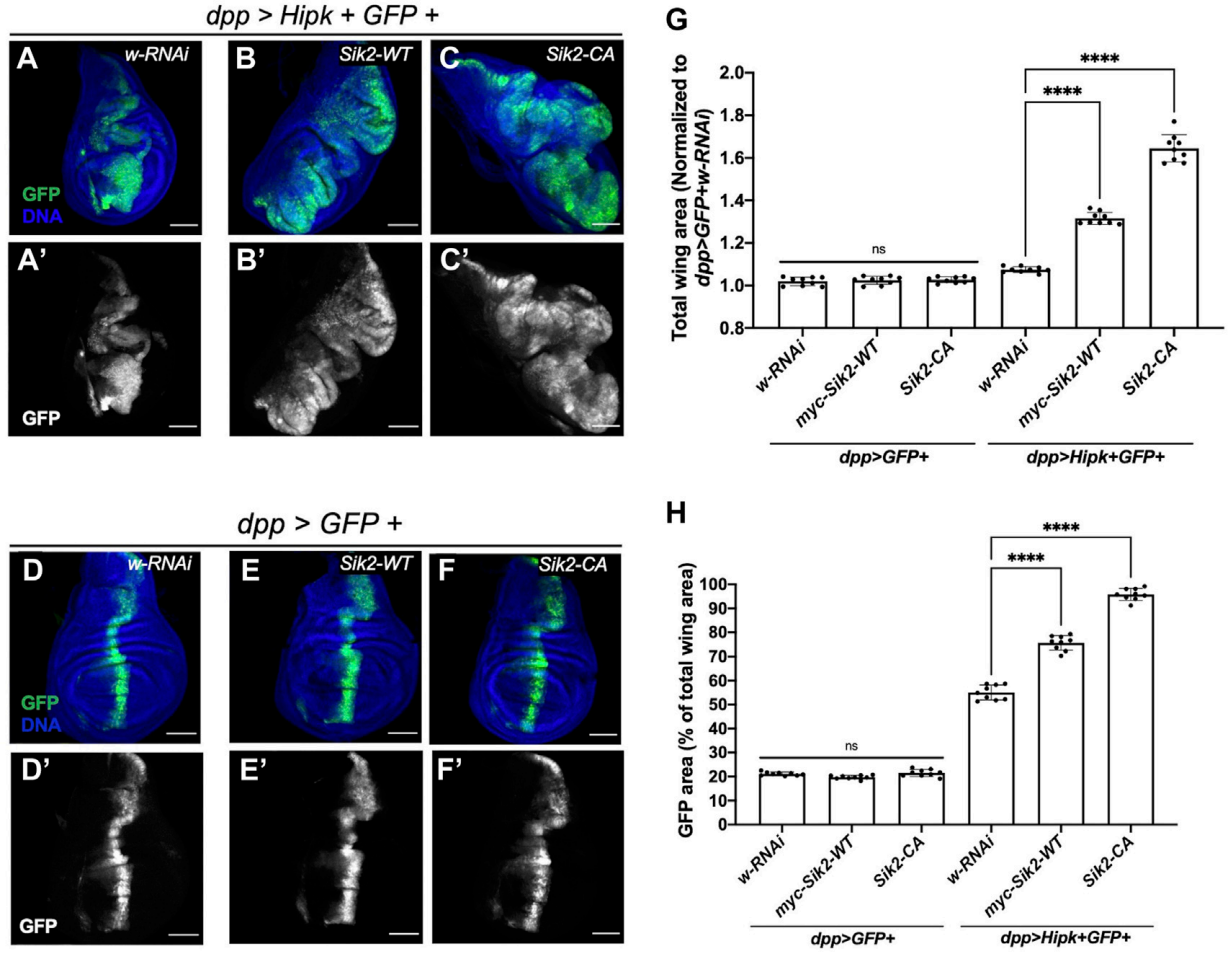
Ectopic expression of Sik2 and Hipk induces significant synergistic overgrowth in third-instar larvae wing imaginal disc tissue **(A–A’–F–F’)** Maximal Z projection representative images of late third-instar larvae (before pupariation) wing imaginal discs of indicated genotypes. GFP (green) indicates the cells expressing the UAS transgene constructs. **(G,H)** Graphs depicting the total wing area and GFP area as a percentage (%) of the total wing area measured using imaging software Fiji. For both graphs, *dpp > GFP + white RNAi* was used to normalise the total wing area. Error bars indicate the standard error of mean (SEM). Statistical analysis included a one-way ANOVA followed by Dunnett’s test to correct for multiple comparisons. *p*-values for the statistical analyses performed correspond to the following symbols: ≥0.0332 (ns), <0.0332 (*), <0.0021(**), <0.0002(***), <0.0001(****). ns = not significant. Scale bars in representative images are 100 µm. N = 9 wing imaginal discs per genotype. This experiment was repeated three times. Flies were raised at 29°C.

We dissected wing imaginal discs from stage matched third instar larvae and found that *dpp > Hipk + GFP* crossed to the *white-RNAi* control and grown at 29°C showed disc hyperplasia which can be seen by the expansion of the GFP-expression domain (Figures 3A, A’) (Blaquiere et al., 2018) relative to control *dpp > GFP + white-RNAi* control (Figures 3D, D’). Quantification of the total disc area shows that Hipk expression alone had a significant effect on overall size with approximately a 10% increase on average, while it had a more dramatic significant effect on area of GFP expressing cells compared to total wing area (Figures 3G, H). When wildtype Sik2 and Hipk were co-expressed, a significantly expanded wing disc size and GFP domain area as a percentage of total wing area could be observed in *dpp > Hipk + Sik2-WT* wing discs (Figures 3B–B’, G, H) as compared to the wing discs expressing Hipk only. Furthermore, enhanced Sik2 activity in *dpp > Hipk + Sik2-CA* discs caused more dramatic tissue overgrowth, with the GFP region occupying more than 90% of the total wing disc compared to approximately 20% in the control discs and approximately 50% in the Hipk overexpression wing discs (Figures 3C–C’, G, H). Thus, the degree of enhancement of the Hipk growth phenotypes is dependent on the activity state of Sik2. We found the same interactions when we looked at the effect of *Sik3-WT* and *Sik3-CA* on *dpp > Hipk* phenotypes (Supplementary Figures S1A–H). Synergy between Hipk and Sik3-CA was particularly pronounced in some discs (Supplementary Figure S1C). The observed hyperplasia is accompanied by a complete loss of normal tissue architecture wherein the GFP region expands into almost all regions of the wing imaginal disc with no visible anterior or posterior compartments (Figures 3C–C’, Supplementary Figures S1B–C’). As stated in the previous results subsection, these larvae of this genotype either die as late L3 larvae or pharates (data not shown). Overexpression of Sik2-WT or Sik2-CA alone using *dpp-Gal4* under these conditions did not cause wing disc tissue overgrowth (Figures 3E–F’), nor did Sik3-WT or Sik3-CA (Supplementary Figures S1E–F’). These results indicate that the extensive and dramatic tumour overgrowth is due to activity-dependent synergistic interactions between Siks and Hipk.

Of note, Sik2-CA has been previously shown to induce adult wing growth using the *hedgehog-Gal4* driver (Wehr et al., 2013). Therefore, we quantified the adult wing *dpp* expression region as a ratio relative to the whole wing area after expression of Sik transgenes. The results are consistent with Wehr et al. in that *dpp > Sik2-WT* did not induce a significant increase in area of *dpp* region compared to control while *dpp > Sik2-CA* significantly increase the ratio of the *dpp* region to the whole wing area (Supplementary Figure S2B–C, F). While Wehr et al. (2013) observed a crumpled wing phenotype with *hh > Sik3-WT* and *hh > Sik3-CA* (see also Supplementary Figure S5), adult wings expressing the Siks in the smaller dpp expression domain resulted in intact adult wings with significantly reduced ratios of the *dpp* area relative to whole wing area and missing the anterior cross vein (Supplementary Figures S2D–F).

### Synergistic activation of the Wnt signalling pathway and induction of dMyc expression and negative regulation of the Hippo signalling pathway in discs overexpressing Siks and Hipk

**FIGURE 4:**
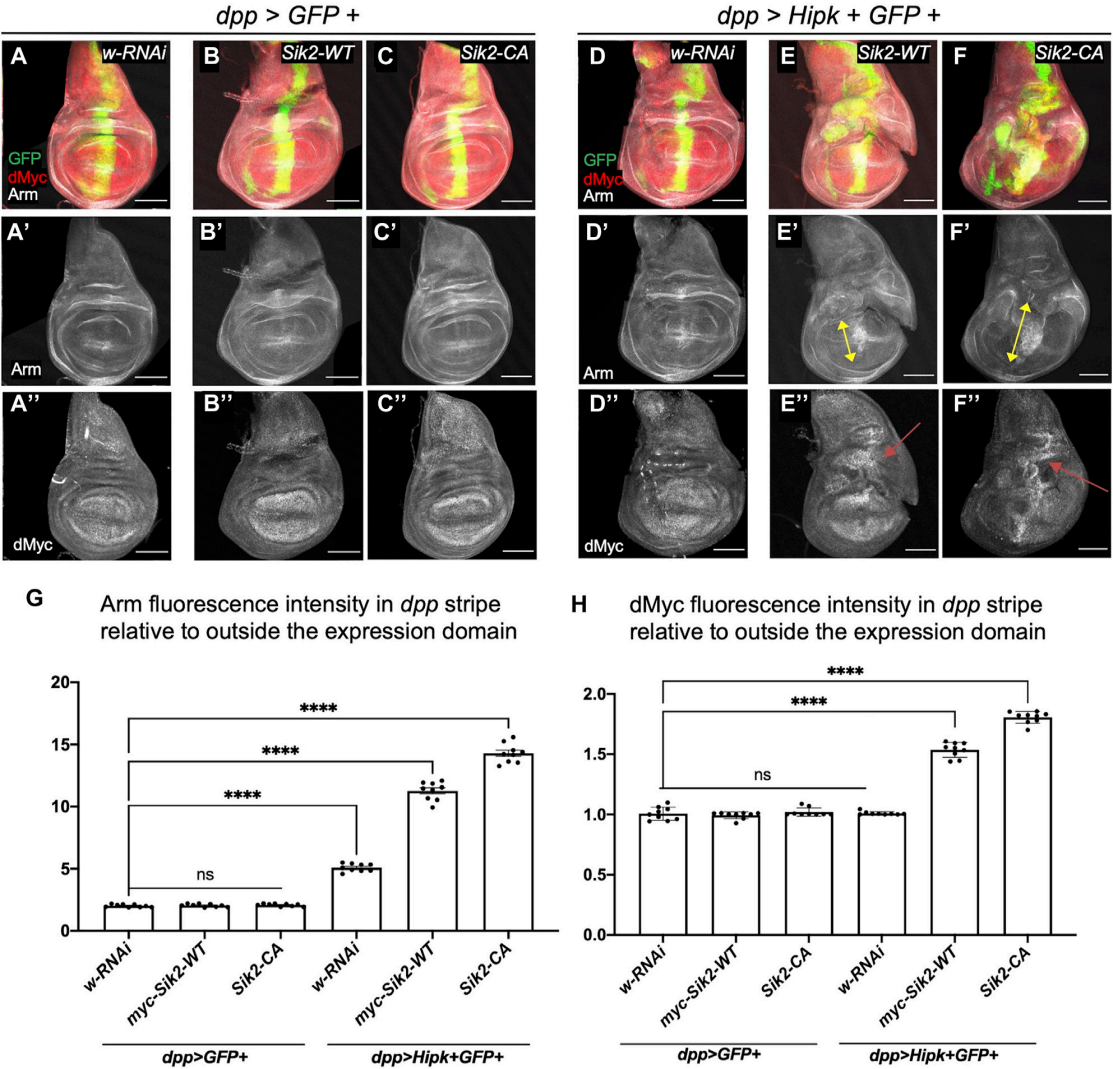
Activation of the Wnt signalling pathway and dMyc in third-instar wing imaginal discs overexpressing Sik2 and Hipk contributes to the significant synergistic overgrowth. Representative images of late third-instar larvae wing imaginal discs of indicated genotypes **(A–A’’–F–F’’)**. GFP (green) indicates the cells expressing the UAS transgene constructs. Wing discs were stained for Wg and dMyc (grayscale). **(G,H)** Graphs depicting the Armadillo and dMyc fluorescence intensity within the dpp region as a ratio to the corresponding fluorescence intensity adjacent and outside the dpp region using software Fiji. For both graphs, *dpp > GFP + white RNAi* was used to normalise the fluorescence intensity. Error bars indicate the standard error of mean (SEM). Statistical analysis included a one-way ANOVA followed by Dunnett’s test to correct for multiple comparisons. *p*-values for the statistical analyses performed correspond to the following symbols: ≥0.0332 (ns), <0.0332 (*), <0.0021(**), <0.0002(***), <0.0001(****). ns = not significant. Scale bars in representative images are 100 µm. N = 9 wing imaginal discs per genotype. This experiment was repeated 3 times. Flies were raised at 25°C.

Hipk can promote numerous signalling pathways, and readouts of pathway activation serve as proxies for Hipk activity. We previously showed that the expression of Hipk leads to stabilization of Armadillo (Arm, *Drosophila* β-catenin) protein and elevated Wnt signaling (Lee et al., 2009; Swarup and Verheyen, 2011). To examine this effect, we grew flies at 25°C where Hipk activity was lower than in previous figures to more clearly detect enhanced signaling readouts, thus the phenotypes of *dpp > Hipk* wing discs are less severe than those shown in Figure 3 (Figure 4D). We next assessed whether expression of Siks either alone or together with Hipk could promote Arm stabilization. Using *dpp-Gal4* to drive expression of either control *white*-*RNAi*, *Sik2-WT* or *Sik2-CA* in wing discs had no effect on Arm protein levels (Figures 4A’–C’, G). In contrast, co-expression of Hipk with Sik2-WT or Sik2-CA led to significant increases in Arm levels within the *dpp* expression domain (arrows in Figures 4E’, F’, G). Similar effects on Arm stabilization were found upon expression of Sik3-WT and Sik3-CA (Supplementary Figures S3A’–F’, G).

Expression of ectopic Hipk promotes several signaling pathways that converge on *dMyc* transcriptional control (Wong et al., 2019). We examined if Sik2 expression could also promote this activity of Hipk. As stated above, under these experimental conditions the effects of Hipk alone are weaker and therefore we do not see elevated dMyc expression in *dpp > Hipk* discs (Figures 4D”, H). Similarly, Sik2-WT and Sik2-CA alone did not cause detectable changes in dMyc protein expression (Figures 4B”–C’’, H). In contrast, co-expression of Sik2-WT and Sik2-CA with Hipk caused significant increases in dMyc levels (Figure 4E”–F”, H). Similar effects on dMyc expression were found upon expression of Sik3-WT and Sik3-CA (Supplementary Figures S3E”, F”, G).

**FIGURE 5:**
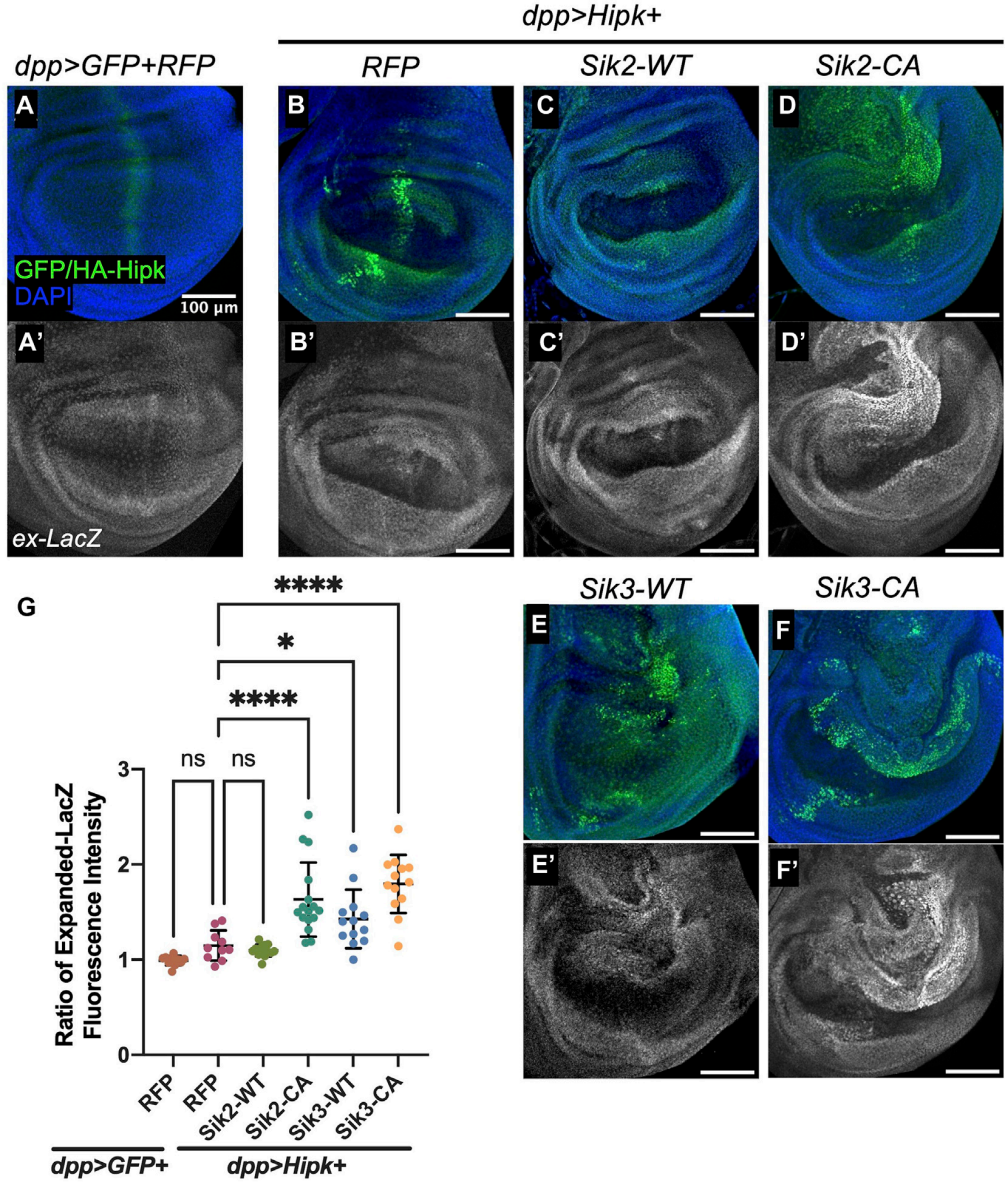
Overexpression of Sik2-CA and Sik3 with Hipk significantly upregulates *expanded-LacZ* fluorescence intensity in third-instar wing imaginal discs **(A–A’–F–F’)** Maximal Z projection representative images of late third-instar larvae (before pupariation) wing imaginal discs of indicated genotypes. FITC (green) indicates the cells overexpressing HA-Hipk. Scale bars in representative images are 100 μm. N = 11, 10, 14, 16, 12 and 13 respectively. This experiment was repeated 4 times. **(G)** Graph depicting Expanded-LacZ fluorescence intensity within the dpp region as a ratio to the corresponding fluorescence intensity adjacent and outside the dpp region using software Fiji. Error bars indicate the standard error of mean (SEM). Statistical analysis included a one-way ANOVA followed by Dunnett’s test to correct for multiple comparisons. *p*-value for the statistical analyses performed correspond to the following symbol: = 0.0498 (*) and <0.0001(****). ns = not significant. Flies were raised at 25°C.

Activated Sik2 and Sik3 have been found to induce wing growth through the Hippo pathway (Wehr et al., 2013). Hipk has also been shown modulate the Hippo pathway by promoting Yorkie (Yki) induced gene expression (Chen and Verheyen, 2012; Poon et al., 2012). We therefore examined the effects on the Yki reporter gene *expanded-lacZ (Ex-lacZ)* under conditions where Hipk and Siks synergize to promote dMyc and Arm. In discs grown at 25°C, we did not see a significantly elevated level of *ex-lacZ* signal in *dpp > Hipk + RFP* wing discs compared to control (Figures 5A–B’, G). Consistent with previous results wherein Hipk and activated Siks have been shown to promote Yki-mediated gene expression individually, we also observed a highly significant increase in *ex-LacZ* fluorescence intensity when both Hipk and activated Siks are co-expressed (Figures 5D–D’, F–F’). While we did not observe a significant enhancement with Sik2-WT (Figures 5C–C’, G), we saw a significant increase following expression of Sik3-WT with Hipk (Figures 5E–E’, G). These results show that Sik2 and Sik3 can both enhance the effect of Hipk on downstream signaling events which may be contributing to tumorigenesis phenotypes.

As we showed that knock-down of endogenous Siks can suppress Hipk-induced leg malformations (Figure 1), we assessed whether this suppression in phenotype was mediated by the Hippo pathway. In 3rd instar leg discs, the *dpp* domain originates from the center of the disc and extends dorsally to the periphery (Abu-Shaar and Mann, 1998). We found that expression of *ex-LacZ* was not significantly changed from levels induced by Hipk alone (Supplementary Figures S4A–A’, D) when endogenous Siks are knocked-down (Supplementary Figures S4B–D). These flies were raised at 29°C, consistent with the experimental conditions in Figure 1. The observation that *ex-lacZ* is not significantly altered in rescued leg discs suggests other targets are responsible for leg defects.

### Siks regulate Hipk-expressing cell area in third instar larvae wing imaginal discs

We have shown that knocking down Siks using RNAi can suppress Hipk-induced leg malformations, and that overexpression of Siks and Hipk induces a significant synergistic overgrowth in the late third-instar larval wing imaginal disc tissue. We therefore wanted to investigate the mechanism by which Siks might regulate Hipk activity. As Hipk protein levels and activity are directly correlated to the level of tissue overgrowth in the eye, wing, and leg imaginal discs (Blaquiere et al., 2018; Wong, Liu, et al., 2020) we investigated whether overexpression of Sik-CAs or knocking down Siks can affect Hipk activity via regulating Hipk protein levels.

**FIGURE 6:**
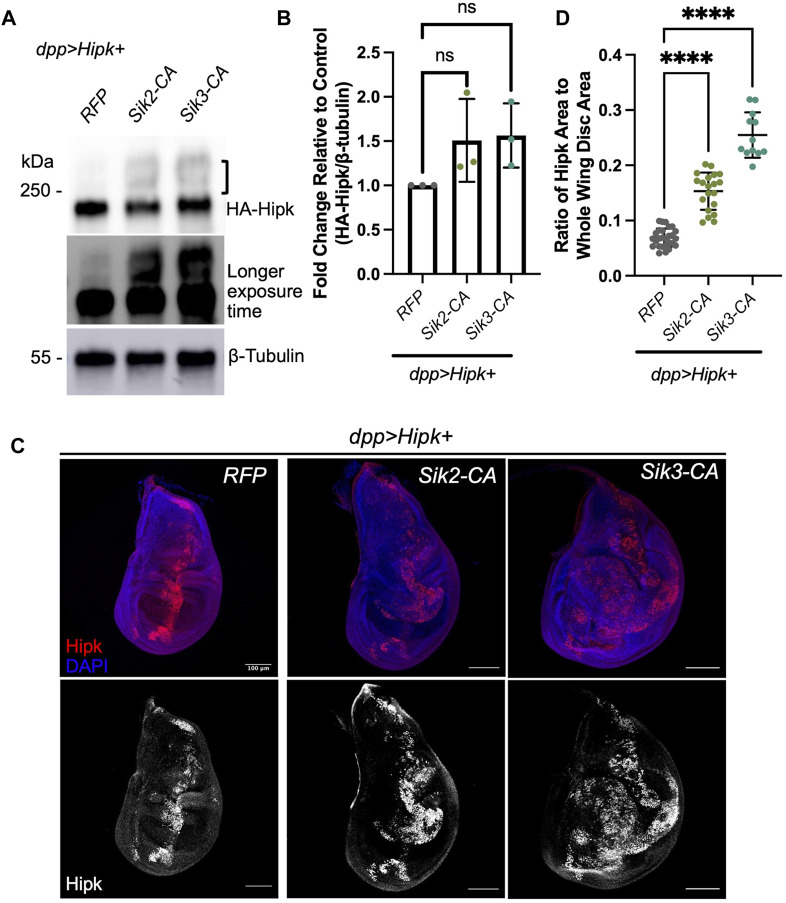
Activated Siks upregulate Hipk protein levels **(A)** Western blot analyses of exogenous Hipk protein levels (top: shorter exposure, bottom: longer exposure) in developmentally stage-matched late third-instar larval head extracts (which included wing imaginal discs) obtained from indicated genotypes. A bracket indicated upshifted Hipk proteins. Anti-β-Tubulin was used as a loading control. Flies were raised at 29°C. **(B)** Graph depicting the HA-Hipk protein band intensity levels relative to the loading control β-Tubulin. HA-Hipk levels of the genotype *dpp > Hipk + RFP* were used to normalize the protein levels. Statistical analysis included a one-way ANOVA followed by Dunnett’s test to correct for multiple comparisons. Values shown are means ± SEM; *n* = 3. **(C)** Developmentally stage-matched late third (L3) instar wing discs of larvae of the indicated genotypes and stained for Hipk (red or white) and DAPI (blue). Scale bars in representative images are 100 µm. N = 27, 20 and 12 respectively. This experiment was repeated 3 times. **(D)** Graph depicting the ratio of Hipk area to whole wing disc area measured using imaging software Fiji. Error bars indicate the standard error of mean (SEM). Statistical analysis included a one-way ANOVA followed by Dunnett’s test to correct for multiple comparisons. *p*-value for the statistical analyses performed correspond to the following symbol: <0.0001(****).

We first examined the levels of exogenous HA-tagged Hipk in developmentally age-matched larval tissue in a variety of genotypes. Endogenous Hipk proteins are undetectable with currently available antibodies and Hipk protein levels are proposed to be tightly regulated by posttranslational modification (Saul and Schmitz, 2013), thus we monitored the effects of Siks on exogenous Hipk. Co-expression of Sik2-CA or Sik3-CA with Hipk (Figures 6A, B) caused a 1.2-2 and 1.2–1.9 fold increase in the levels of Hipk protein, respectively, indicating that overexpression of constitutively active Siks can increase Hipk protein levels. However, this positive trend is not statistically significant. We observed that Hipk proteins had altered migration in the presence of either Sik2-CA or Sik3-CA, indicating increased post-translational modifications (indicated by a bracket in Figure 6A). In a longer exposure, a low level of upshift is also seen in *dpp > Hipk + RFP* extracts.

Due to the positive trend in Hipk protein levels from immunoblotting, we wanted to see if Hipk protein was also more abundant in wing imaginal discs. Since co-expression of Hipk and Siks led to dramatic increases in disc size, we quantified the area of fluorescence corresponding to the cells in which these transgenes are expressed using an antibody that detects exogenous Hipk. Endogenous levels of Hipk are undetectable at this resolution. Analysis of the ratio of Hipk-expressing area to the total area of the whole wing imaginal disc indicates that overexpression of both Sik2-CA and Sik3-CA can significantly increase the ratio of Hipk-expressing area compared to control (Figures 6C, D), consistent with our quantification of GFP-expressing cells in Figure 3.

**FIGURE 7:**
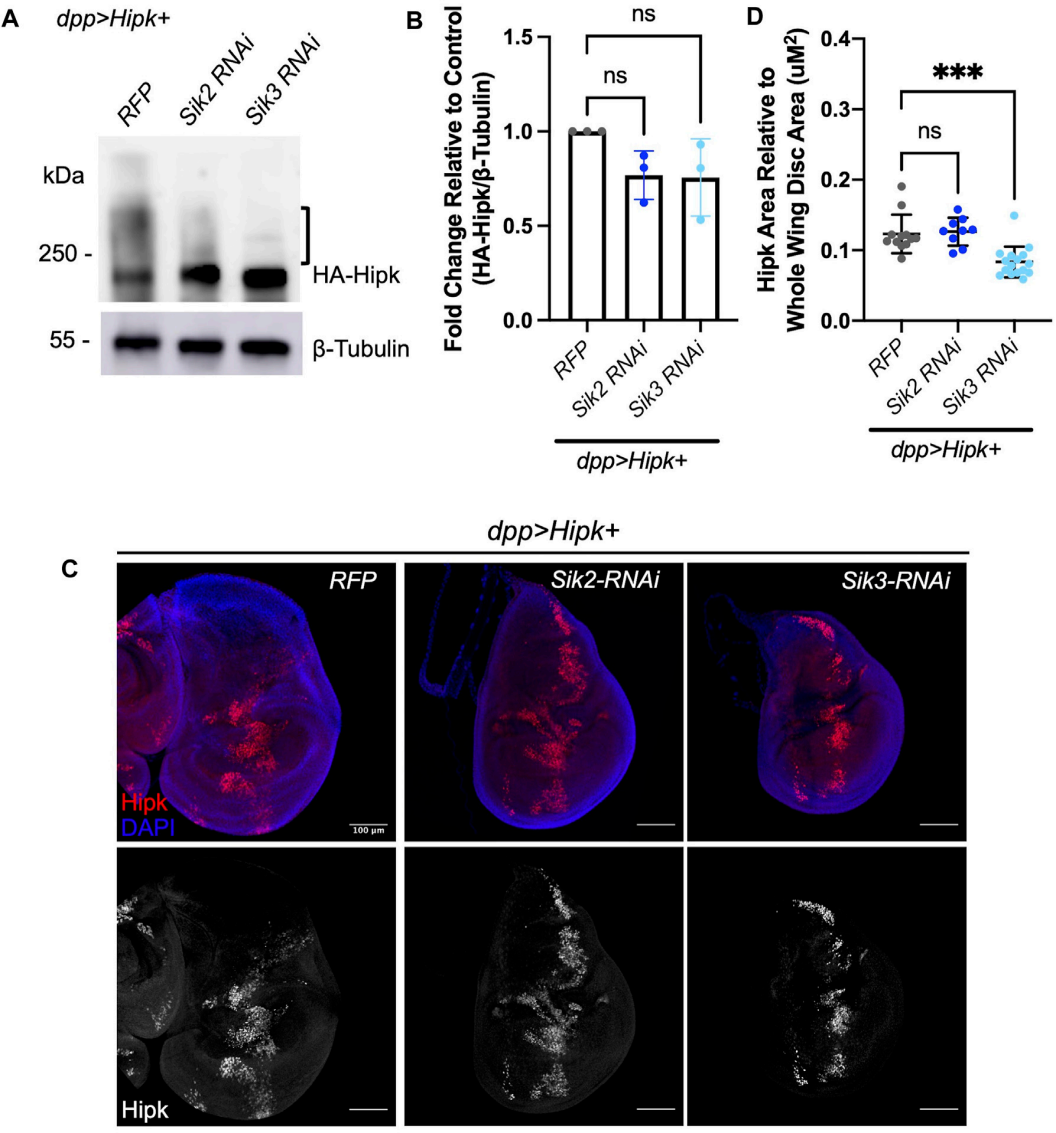
Depletion of endogenous Siks downregulates Hipk protein levels **(A)** Western blot analyses of exogenous Hipk protein levels in developmentally stage-matched late third-instar larval head extracts (which included wing imaginal discs) obtained from indicated genotypes. Anti-β-Tubulin was used as a loading control. Flies were raised at 29°C. **(B)** Graph depicting the HA-Hipk protein band intensity levels relative to the loading control β-Tubulin. HA-Hipk levels of the genotype *dpp > Hipk + RFP* were used to normalize the protein levels. Statistical analysis included a one-way ANOVA followed by Dunnett’s test to correct for multiple comparisons. Values shown are means ± SEM; *n* = 3. **(C)** Developmentally stage-matched late third (L3) instar wing discs of larvae of the indicated genotypes and stained for Hipk (red or white) and DAPI (blue). Scale bars in representative images are 100 µm. N = 12, 9 and 16 respectively. This experiment was repeated 3 times. **(D)** Graph depicting the ratio of Hipk area to whole wing disc area measured using imaging software Fiji. *Sik3-RNAi BDRC #28366* was used. Error bars indicate the standard error of mean (SEM). Statistical analysis included a one-way ANOVA followed by Dunnett’s test to correct for multiple comparisons. *p*-values for the statistical analyses performed correspond to the following symbols: ≥0.0332 (ns) and <0.0002(***). ns = not significant.

To complement our overexpression studies, we examined the effects on Hipk protein levels following depletion of *Sik2* and *Sik3*. Depletion of endogenous *Sik2* and *Sik3* via RNAi (Figures 7A, B) caused a 0.6–0.8 and 0.5–0.9 fold reduction in Hipk protein levels (Figure 7B), relative to expression of Hipk combined with the *RFP* control though this trend is not statistically significant. In these analyses we observed reduced Hipk migration upshifts, indicative of decreased post-translational modifications. Analysis of the ratio of Hipk-expressing cells to the total area of cells in the whole wing imaginal disc indicates that endogenous knockdown of Sik3 can significantly decrease the number of Hipk-expressing cells compared to control (Figures 7C, D), which likely reflects the supressed phenotype we observe in Figure 1.

### Siks interact with Hipk

**FIGURE 8:**
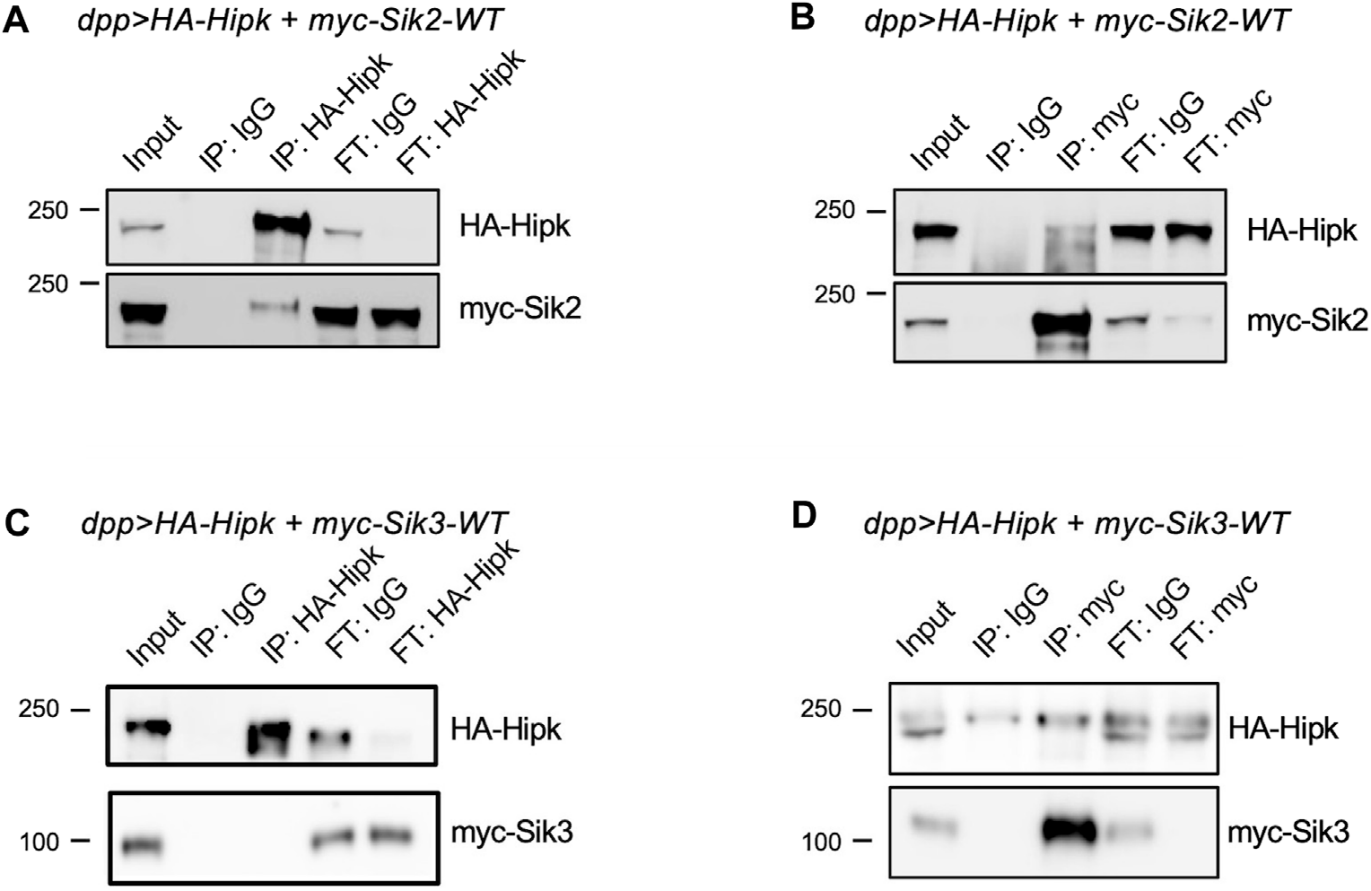
Sik2 and Sik3 can interact with Hipk protein **(A,B)** HA-Hipk and myc-Siks pull-down assays using protein lysates extracted from *dpp> HA-Hipk + myc-Sik2WT* and *dpp> HA-Hipk + myc-Sik3WT* **(C,D)**, shown as the protein lysate extracts (Input), and the eluates (IP) and the flow-throughs (FT). The protein lysates were incubated with either IgG, anti-Hipk or anti-myc antibodies **(A–C)** or IgG, myc-tagged agarose beads **(D)** followed by co-immunoprecipitation. Western blot showing pull-down of myc-Sik2 with HA-Hipk **(A)** as well as the reciprocal IP showing that HA-Hipk was pull-down with myc-Sik2.

As we have shown that Siks can potentially regulate Hipk protein levels, we wondered if Siks were able to physically interact with Hipk. We expressed HA-Hipk and myc-tagged Sik proteins using *dpp-Gal4* and subjected protein extracts to reciprocal co-immunoprecipitation (IP) assays. We first found that anti-Hipk antibodies could pull down myc-Sik2 (Figure 8A, IP:HA-Hipk), while control IgG agarose did not pull down detectable amounts of Hipk or Sik2 (Figure 8A, IP:IgG). Conversely, anti-myc antibody was able to pull down both myc-Sik2 and Hipk (Figure 8B, IP:myc). We also tested the interaction between Hipk and Sik3 (Wehr et al., 2013). Using anti-Hipk antibodies, we pulled down Hipk and under these experimental conditions Hipk and Sik3 interactions were not detectable (Figure 8C, IP:IgG). Conversely, reciprocal co-IP using myc agarose beads to precipitate myc-Sik3 did reveal an enriched interaction with Hipk compared to control agarose (Figure 8D, IP:myc) indicating that Hipk and Sik3 also interact physically.

### Overexpressed Sik2-CA cannot induce overgrowth in absence of Hipk

We have shown that depletion of endogenous Siks suppresses Hipk-induced leg malformations, that co-expression of Siks can synergistically enhance Hipk-induced phenotypes and potentially affect levels of Hipk protein and degrees of Hipk posttranslational modifications, and that Siks and Hipk can interact physically. These results suggest a model in which endogenous Siks are needed for the activity of exogenous Hipk. An alternative model could posit that Siks are acting downstream of Hipk and that loss of Siks therefore suppresses Hipk-induced overgrowth. To address the second model, we tested whether depletion of endogenous Hipk could modulate Sik induced phenotypes.

**FIGURE 9:**
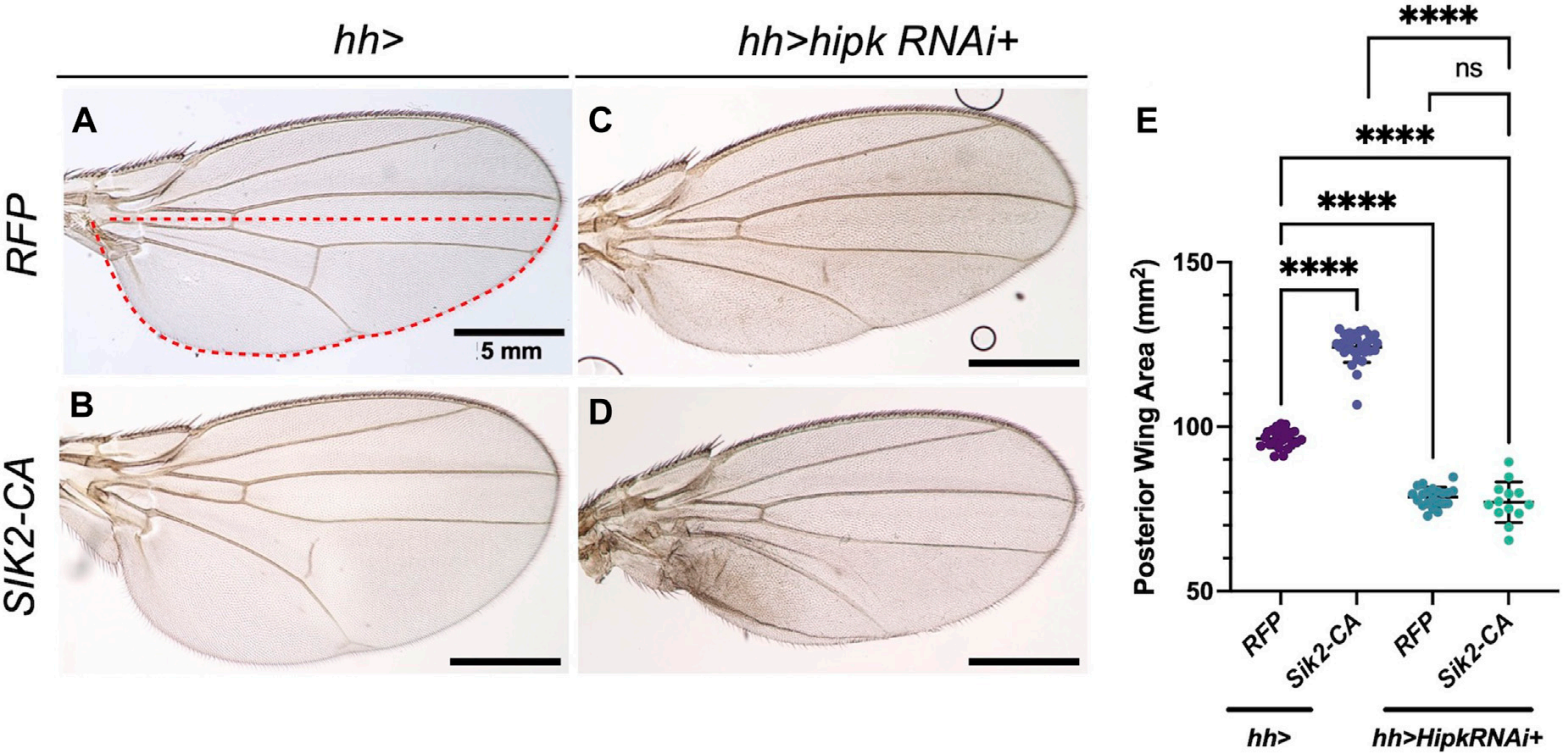
Overexpression of Sik2-CA cannot induce overgrowth after depletion of endogenous Hipk **(A–D)** Adult *Drosophila* wings of the indicated genotypes with the *hh-Gal4* expression domain in red dashed line. Scale bars in representative images are 5 mm. N = 24, 30, 21 and 13 respectively. This experiment was repeated 4 times. **(E)** Graph depicting the posterior wing area measured using imaging software Fiji. Error bars indicate the standard error of mean (SEM). Statistical analysis included a one-way ANOVA followed by Dunnett’s test to correct for multiple comparisons. *p*-value for the statistical analyses performed correspond to the following symbol: <0.0001(****). ns = not significant. Flies were raised at 25°C.

Wehr et al. (2013) showed that *hh > Sik2-CA* can induce a significant increase in the area of posterior wing blade compared to a control wing (Figures 9A, B, E). We used this phenotype as a starting point for testing the effect of *hipk* depletion. Endogenous depletion of Hipk alone using *hh > hipk-RNAi* significantly reduced the posterior area (Figures 9C, E). Co-expression of Sik2-CA with *hipk-RNAi* did not result in a significant change of the posterior area compared to depletion of *hipk* alone (Figures 9D, E), showing that Sik2-CA is not able to induce growth after Hipk depletion.

In contrast to Sik2-CA, Wehr et al. (2013) showed that expression of Sik3-CA induced severe malformations resulting in various crumpled wing phenotypes (Supplementary Figures S5A–C). We show that depletion of Hipk in the *hh > Sik3-CA* background did not noticeably affect the range of Sik3-CA induced phenotypes. In both genotypes we found a range of phenotypes represented in (Supplementary Figures S5A–F). Given the nature of the wing defects, we were not able to quantify if *hipk-RNAi* had any significant effects. From these results we cannot rule out that Hipk may also affect Sik activity, though further studies will be needed to address this more fully.

## Discussion

*Drosophila* tumor models have been used to study a number of complex synergistic interactions. Using biochemical and genetic analyses, we were able show that Hipk and Siks interact, and Siks augment Hipk tumorigenic activity. Many types of cancer involve heterogenous alterations of multiple genes affecting multiple pathways, which therefore can rapidly develop resistance to a single line of treatment. Hence, understanding tumor biology through molecular and genetic studies will potentially lead to breakthroughs in future treatments by targeting Hipk and Sik as novel targets.

### Developmental delay as a result of synergistic interactions

We find that *Drosophila* larvae containing epithelial tumors induced by co-expression of Hipk and Sik-CAs entered metamorphosis 3.5-6 days after control larvae (Figure 2) and in particular, a significant portion of larvae expressing Hipk and Sik3-CA are unable to pupariate and die as larvae (data not shown). During the prolonged larval phase, larvae become progressively larger in size (Figure 2B) compared to control. This whole-body phenotype that is distant from the tumor site is also observed in other *Drosophila* tumor models (Bilder et al., 2021). Mutation of “lethal-giant”, a tumor-suppressor gene results in developmentally delayed larvae that die before pupariation (Hadorn, 1937). Larval development in *Drosophila* relies on conserved hormonal and metabolic homeostasis, such as insulin-like peptides and steroids, indicating that this significant developmental delay is a result of hormonal imbalance and metabolic dysregulation (Garelli et al., 2012; Kwon et al., 2015). Further studies will be needed to determine the mechanism by which Hipk and Siks induce these effects.

### A novel interaction between Hipk and Siks in the context of tumorigenesis

We showed that Hipk co-expression with Siks induces significant synergistic overgrowth in the *Drosophila* larval wing imaginal disc (Figure 3). In human cancer patients, heterogeneity within the tumor cell population is well-established and these heterogenous cancer cells with multiple mutations interact with each other and their tumor microenvironment to result in cancer progression (Turajlic et al., 2019). The genetic tools available for the fruit fly allow us to overexpress our oncogenic proteins of interest in a subset of the *Drosophila* cell population and subsequently study the synergistic effects of Hipk and Siks in the tumor microenvironment. Previously, it has been shown that interaction between oncogenic protein Ras^V12^ and loss of function tumor suppressor *scribbled* is required for tumor development in *Drosophila* eye-antennal discs (Pagliarini and Xu, 2003; Wu et al., 2010). In addition, it has also been shown that induced activation of oncogenic Ras and Src in the *Drosophila* eye epithelia resulted in small benign tumors, however when co-expressed with Sik2-CA resulted in tumor overgrowth (Hirabayashi and Cagan, 2015). A study by Şahin et al. (2020) showed complex genetic interactions including potent synergy of Sik2 and Sik3 with a Notch-based tumor model. These results demonstrate that like the results of this study, different oncogenic mutations can synergise to produce tumor overgrowths.

In addition, we also demonstrated that Sik2-WT itself does not induce tumorigenesis in the larval wing imaginal disc or the adult *Drosophila* wing (Figure 3, Supplementary Figure S2). This is consistent with previous research, wherein overexpressing Sik2-WT in the entire posterior compartment of the adult *Drosophila* wing did not result in significant posterior compartment growth. Using the same driver, a significant posterior compartment growth was seen with Sik2-CA (Wehr et al., 2013) while in our experiments we expressed Sik2-CA in a very narrow region (∼20%) straddling the anterior-posterior boundary of the disc instead of the entire posterior compartment and did not see a significant change (Figures 3D–H). However, in adult wings we did see a significant increase in the dpp region from 20% in the control to ∼23% with Sik2-CA overexpression (Supplementary Figure S2). It is worth noting that when Sik2-CA is expressed in the posterior compartment of the adult wing, the ratio of the posterior compartment to anterior was expanded from 55%–60%–65% (Wehr et al., 2013). In our results, overexpression of Sik2-CA with Hipk resulted in expansion of the dpp region from 20% in the control to ∼90%, further demonstrating the striking synergy between Sik and Hipk.

We found that the Wnt and dMyc signalling pathways were upregulated while the Hippo pathway was negatively regulated by overexpression of Hipk and Siks (Figures 4, 5). Activation of the Wnt signalling pathway is a frequent occurrence in many cancer types, and results in cancer cells with stem-cell like properties of sustained growth (Bugter et al., 2021). Upregulation of Myc and Myc-related pathways occur in the vast majority of cancers and has wide-ranging effects on the cancer cell programmes and tumor microenvironment (Dhanasekaran et al., 2022). Finally, deregulation of the Hippo pathway is present in various human cancers including lung, colorectal, liver and prostate cancers and has been shown to result in cell proliferation, cell survival, metastasis and cell regeneration (Harvey et al., 2013). Both Siks and Hipks can promote Yorkie-mediated gene expression, though through distinct mechanisms. Siks are proposed to modulate the upstream negative regulation of Yki, while Hipk was shown by us and others to enhance Yorkie activity itself (Chen and Verheyen, 2012; Wehr et al., 2013). It is not yet clear whether Sik also promotes Hipk in this context is since depletion of Sik can rescue Hipk-induced leg overgrowths, but not by significantly altering levels of *ex-lacZ* expression. Therefore, the conservation of signalling pathways in the *Drosophila* could provide valuable insight into how Hipk and Sik can induce tumorigenesis in human cancer patients as well.

### Siks as regulators of Hipk

The results presented in this study are consistent with a model in which Siks can act upstream of Hipk to modulate their ability to induce tumorigenic phenotypes. This hypothesis emerged from a combined examination of genetic interactions with biochemical evidence that Hipk protein levels and posttranslational modification are potentially altered upon modulation of Sik2 and Sik3.

We also show that Sik-CAs have a more synergistic tumorigenesis effect when co-expressed with Hipk than Sik-WT (Figure 2), suggesting that the kinase function of Siks is necessary for this synergistic tumorigenesis. Both Sik2-CA and Sik3-CA are mutant for a regulatory PKA site, which abolishes or reduces 14-3-3 binding. This amino acid change inhibits PKA regulation of SIK activity, resulting in insensitivity to cAMP signaling and constitutive SIK kinase activity (Okamoto et al., 2004; Wehr et al., 2013; Sonntag et al., 2018). SIKs have been shown to control the phosphorylation of transcription factors such as histone deacetylases (HDAC IIs) and cAMP-regulated transcriptional coactivators (CRTCs), in turn changing the nuclear-cytoplasmic localisation of these SIK substrates (Wein et al., 2018). Our Western blot data detects a trend in which more Hipk protein is observed when Sik-CAs are co-expressed and less Hipk protein when endogenous Siks are knocked down. The area of Hipk-expressing cells detected in the wing imaginal disc is significantly increased when co-expressed with Sik-CAs, further supporting this model. Since our protein blots were performed with whole heads, including tissues not expressing Hipk, the total levels of protein could be underrepresented in the blots compared to examination of just wing discs. In addition, our Western blot data shows Sik-CAs induced an upshift of the exogenous Hipk band (Figure 6A), suggesting that Sik-CAs are inducing post-translational modification of HA-Hipk. This HA-Hipk upshift is not visible when endogenous Sik3 is knocked down via RNAi (Figure 7A), further suggesting that endogenous Siks can promote post-translational modification of HA-Hipk either directly or indirectly.

Based on our findings, we hypothesize that Siks act on Hipk by direct phosphorylation and activation. However, alignment of the Hipk protein sequence to a canonical SIK substrate phosphorylation motif LXB(S/T)XSXXXL (B, basic residue; X, any residue) (Horike et al., 2003; Okamoto et al., 2004; Sonntag et al., 2018) revealed no consensus motif in Hipk. Notably, multiple physiologically important SIK substrates also do not align with this motif. *In vitro* studies using recombinant SIK1 have shown that while SIK1 phosphorylates CREB-specific coactivator TORC2 (also known as CRTC2) at conserved serines, the surrounding motif does not align with the canonical motif (Uebi et al., 2010). In *Drosophila*, the cAMP-regulated transcriptional coactivator CRTC has been identified as a direct target of Sik2, and the conserved S157 in CRTC as the phosphorylation site for Sik2 (Choi et al., 2011). However, the surrounding peptide does not align with the canonical phosphorylation motif. In another example, biochemical and genetic assays in *Drosophila* have shown that Sik3 interacts with G6PD, and thereby regulates glucose metabolism (Teesalu et al., 2017). The canonical SIK motif is also not found in G6PD. It has been proposed that an alternative phosphorylation motif may be present in SIK targets (Sonntag et al., 2018). Using bioinformatics prediction models, several potential phosphorylation sites in Hipk have been identified. Further studies with purified recombinant proteins will be necessary to reveal whether Hipk possesses a variant SIK recognition motif.

An alternative or additional mechanism that could be at play suggests that Hipk can modulate Sik activity. In this case, Hipk might act upstream of Sik. While some of our genetic interaction studies could be interpreted to support this model, we suggest this could be in addition to regulation of Hipk by Sik, rather than instead of. Further studies will more deeply investigate whether Siks could also be regulated by Hipk.

## Data Availability

The original contributions presented in the study are included in the article/Supplementary Material, further inquiries can be directed to the corresponding author.
