# Supplementary material for: The AMPK-like protein kinases Sik2 and Sik3 interact with Hipk and induce synergistic tumorigenesis in a Drosophila cancer model
Source: Front Cell Dev Biol. 2023 Oct 3;11:1214539. doi: 10.3389/fcell.2023.1214539 (PMC10579798; doi:10.3389/fcell.2023.1214539)

“The AMPK-like protein kinases Sik2 and Sik3 interact with Hipk and induce synergistic tumorigenesis in a Drosophila cancer model”

Kewei Yu[1,2,] Niveditha Ramkumar[1,2,] Kenneth Kin Lam Wong1[,2†], Gritta Tettweiler[1,2] and Esther M. Verheyen[1,2*]
[1]Department of Molecular Biology and Biochemistry, Simon Fraser University, Burnaby, BC, Canada, [2]Centre for Cell Biology, Development and Disease, Simon Fraser University, Burnaby, BC, Canada

Supplementary Figure Legends

Fig. S1. Ectopic expression of Sik3 and Hipk induces significant synergistic overgrowth in third-instar larvae wing imaginal disc tissue
Maximal Z projection representative images of late third-instar larvae (before pupariation) wing imaginal discs of indicated genotypes (A-A’-F-F’. GFP (green) indicates the cells expressing the UAS transgene constructs. (G-H) Graphs depicting the total wing area and GFP area as a percentage (%) of the total wing area measured using imaging software Fiji. For both graphs, *dpp > GFP + white RNAi* was used to normalise the total wing area. Error bars indicate the standard error of mean (SEM). Statistical analysis included a one-way ANOVA followed by Dunnett’s test to correct for multiple comparisons. *P*-values for the statistical analyses performed correspond to the following symbols: ≥0.0332 (ns), <0.0021(**), < 0.0001(****). ns=not significant. Scale bars in representative images are 100 μm. N=9 wing imaginal discs per genotype. This experiment was repeated three times. Flies were raised at 29°C.

Supplementary Fig. 1

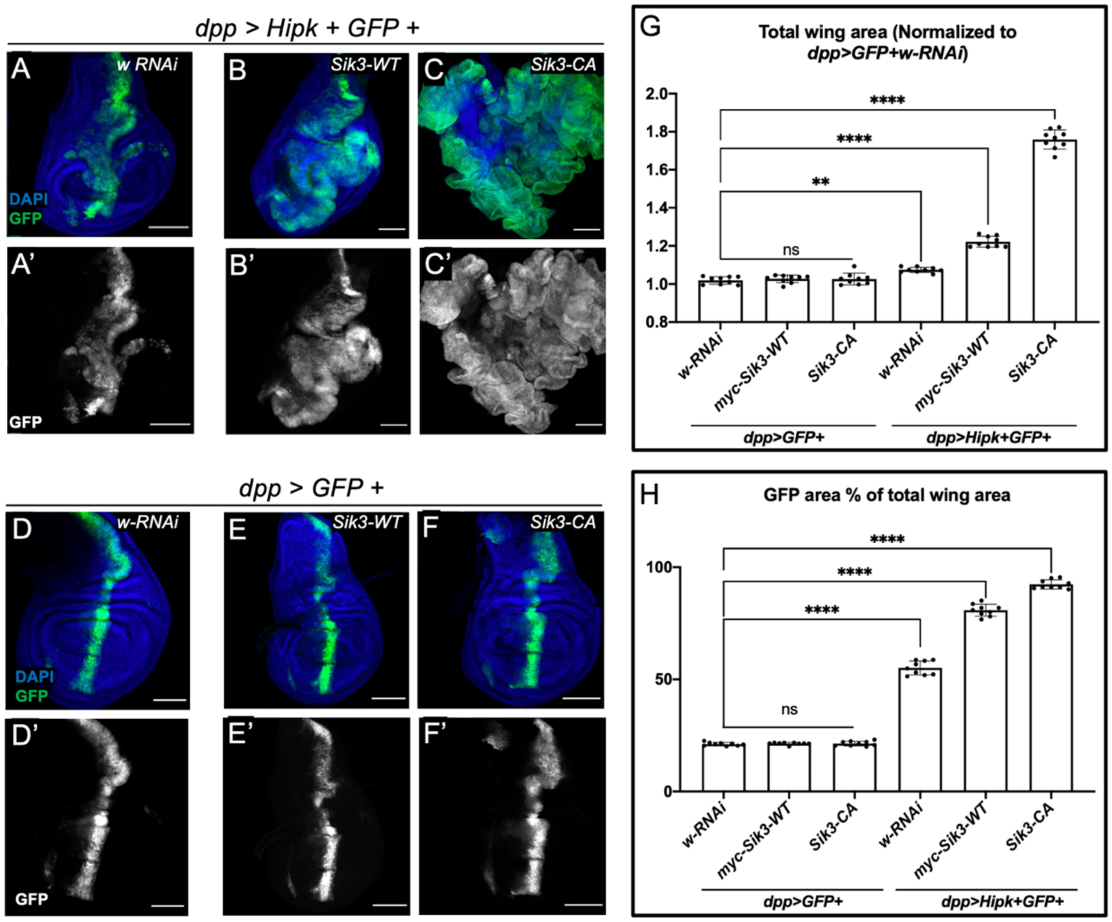

Fig. S2 Ectopic expression of Sik2-CA increase adult wing growth while Sik3-WT and Sik3-CA decrease adult wing growth

(A-E) Adult drosophila wings of the indicated genotypes with the *dpp-Gal4* expression domain in red dashed line. Scale bars in representative images are 5 mm. N=15, 25, 14, 24 and 26 respectively. This experiment was repeated 1 time. (F) Graph depicting the ratio of dpp area to whole wing area measured using imaging software Fiji. Error bars indicate the standard error of mean (SEM). Statistical analysis included a one-way ANOVA followed by Dunnett's test to correct for multiple comparisons. *P*-value for the statistical analyses performed correspond to the following symbol: < 0.0001(****). Flies were raised at 29°C.

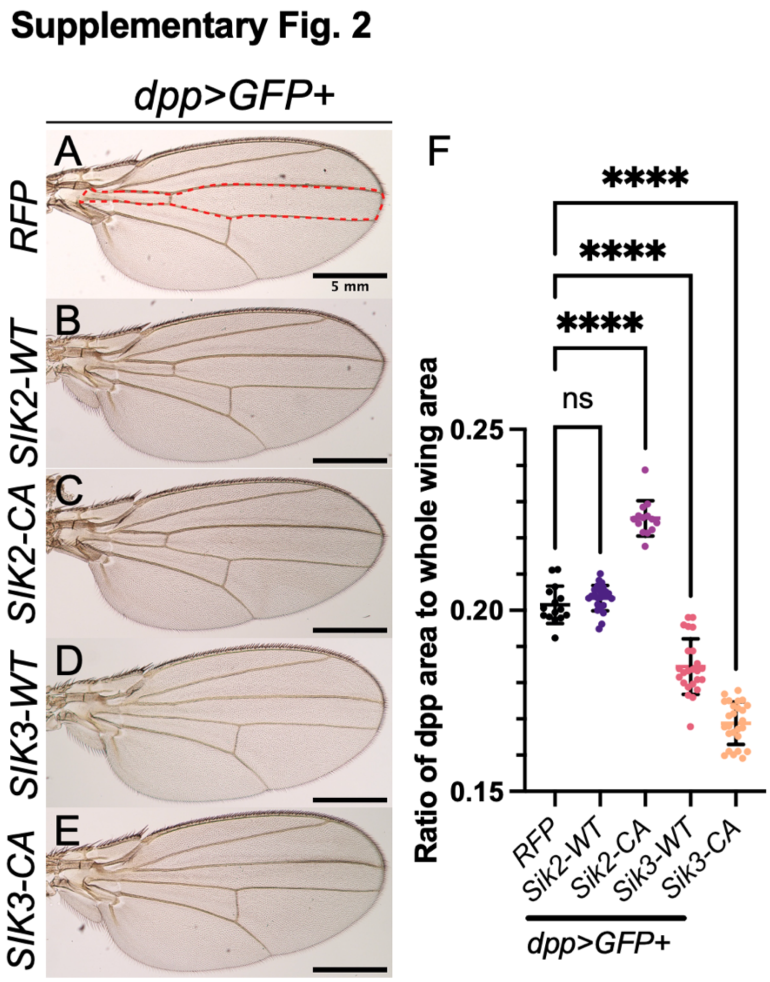

Fig. S3. Activation of the Wnt signalling pathway and dMyc in third-instar wing imaginal discs overexpressing Sik3 and Hipk contributes to the significant synergistic overgrowth Representative images of late third-instar larvae wing imaginal discs of indicated genotypes (A-A''-F-F''). GFP (green) indicates the cells expressing the UAS transgene constructs. Wing discs were stained for Wg and dMyc (grayscale). (G-H) Graphs depicting the Armadillo and dMyc fluorescence intensity within the dpp region as a ratio to the corresponding fluorescence intensity adjacent and outside the dpp region using software Fiji. For both graphs, *dpp > GFP + white RNAi* was used to normalise the fluorescence intensity. Error bars indicate the standard error of mean (SEM). Statistical analysis included a one-way ANOVA followed by Dunnett's test to correct for multiple comparisons. *P*-values for the statistical analyses performed correspond to the following symbols: ≥0.0332 (ns), < 0.0001(****). ns=not significant. Scale bars in representative images are 100 µm. N=9 wing imaginal discs per genotype. This experiment was repeated 3 times. Flies were raised at 25°C.

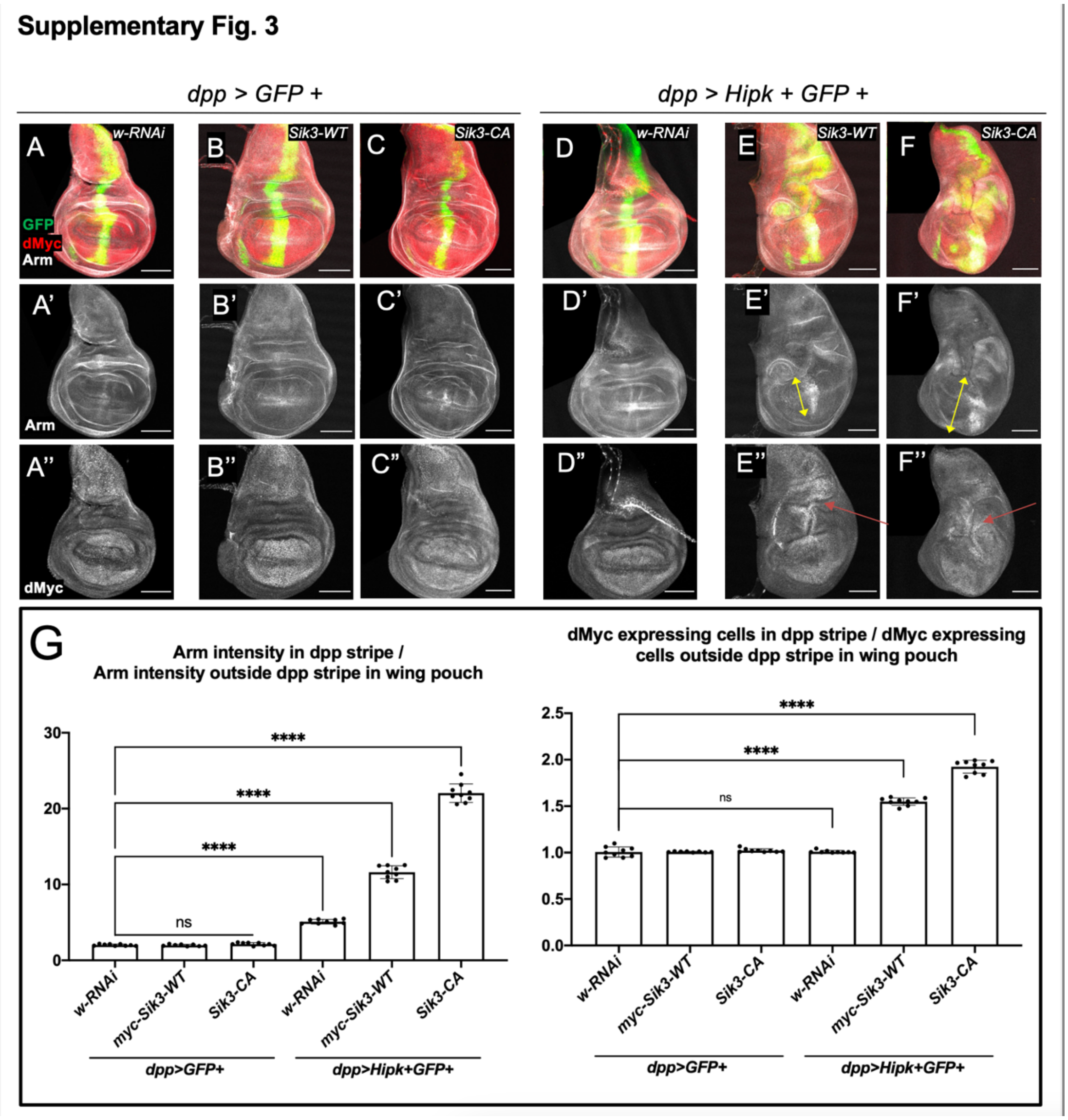

Fig. S4 Depletion of Endogenous Siks has no significant effect on Hipk-induced *expanded-LacZ* fluorescence levels
(A-C) Maximal Z projection representative images of late third-instar larvae (before pupariation) wing imaginal discs of indicated genotypes (A-A'-C-C'). Sik3-RNAi BDRC #28366 was used. FITC (green) indicates the cells overexpressing HA-Hipk. Scale bars in representative images are 100 µm. N=9, 10 and 11 respectively. This experiment was repeated 1 time. (D) Graph depicting Expanded-LacZ fluorescence intensity within the dpp region as a ratio to the corresponding fluorescence intensity adjacent and outside the dpp region using software Fiji. Error bars indicate the standard error of mean (SEM). Statistical analysis included a one-way ANOVA followed by Dunnett's test to correct for multiple comparisons. ns=not significant. Flies were raised at 29°C.

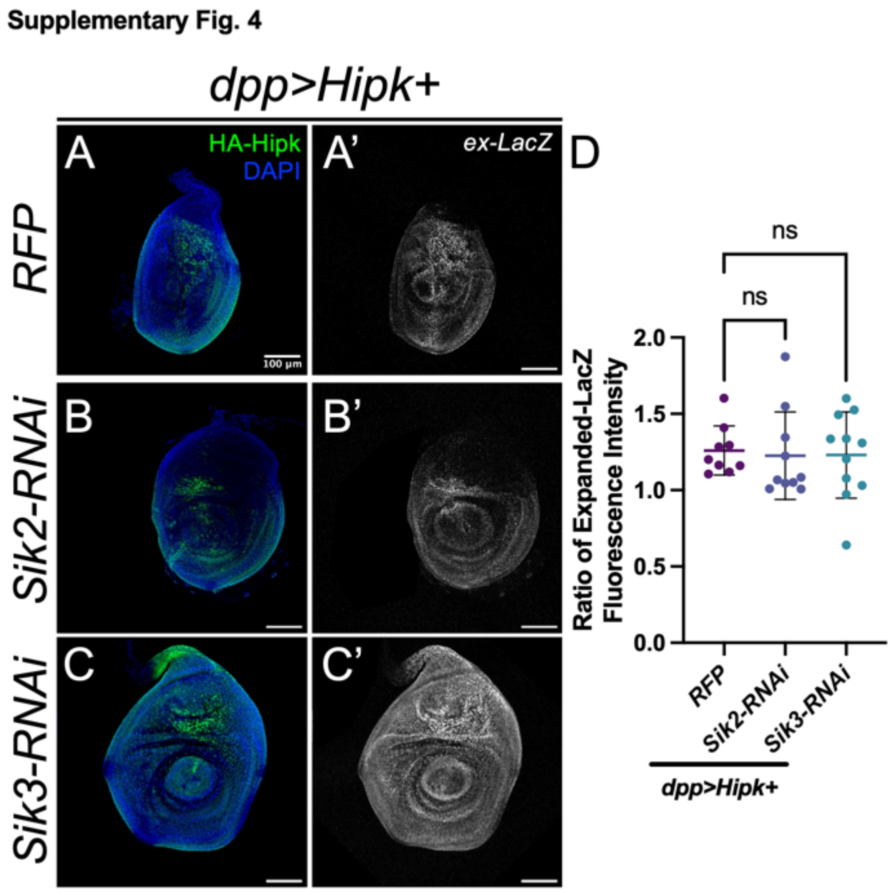

Fig. S5 Knock down of endogenous Hipk has no noticeable effect on the crumpled wing phenotype induced by ectopic expression of Sik3-CA

(A-F) Representative adult Drosophila wings of the indicated genotypes. Scale bars in representative images are 5 mm. N=11 and 6 respectively. This experiment was repeated 1 time. Flies were raised at room temperature.

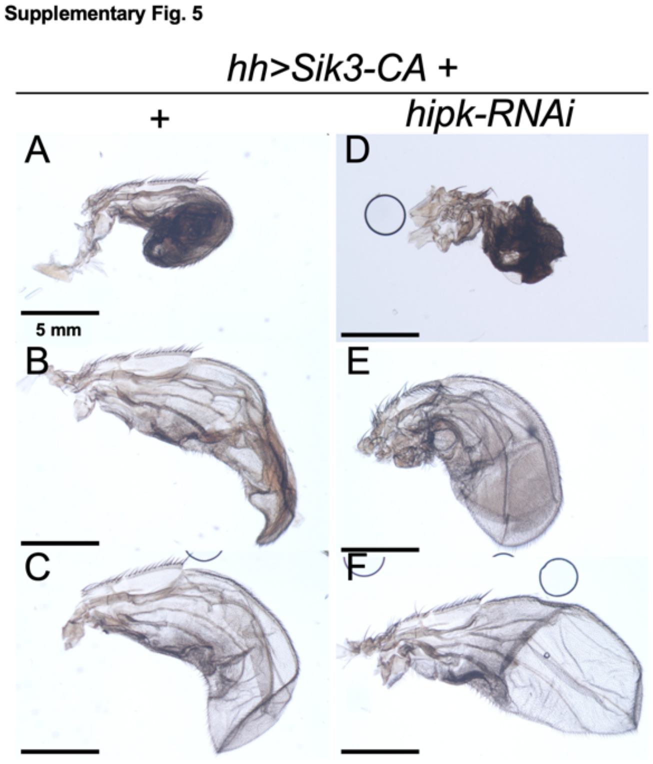

Supplement: Supplementary file 1 [file DataSheet1.pdf]
